# One-Stage vs. Two-Stage Brachio-Basilic Arteriovenous Fistula for Dialysis Access: A Systematic Review and a Meta-Analysis

**DOI:** 10.1371/journal.pone.0120154

**Published:** 2015-03-09

**Authors:** Khalid Bashar, Donagh A. Healy, Sawsan Elsheikh, Leonard D. Browne, Michael T. Walsh, Mary Clarke-Moloney, Paul E. Burke, Eamon G. Kavanagh, Stewart R. Walsh

**Affiliations:** 1 Department of Vascular Surgery, University Hospital Limerick, Limerick, Ireland; 2 Department of Acute Medicine, James Connolly Memorial Hospital, Dublin, Ireland; 3 Centre for Applied Biomedical Engineering Research (CABER), Department of Mechanical, Aeronautical & Biomedical Engineering, Materials and Surface Science Institute, University of Limerick, Limerick, Ireland; 4 Department of Surgery, National University of Ireland, Galway, Ireland; Medical University of Graz, AUSTRIA

## Abstract

**Introduction:**

A brachiobasilic arteriovenous fistula (BB-AVF) can provide access for haemodialysis in patients who are not eligible for a more superficial fistula. However, it is unclear whether one- or two-stage BB-AVF is the best option for patients.

**Aim:**

To systematically assess the difference between both procedures in terms of access maturation, patency and postoperative complications.

**Methods:**

Online search for randomised controlled trials (RCTs) and observational studies that compared the one-stage versus the two-stage technique for creating a BB-AVF.

**Results:**

Eight studies were included (849 patients with 859 fistulas), 366 created using a one-stage technique, while 493 in a two-stage approach. There was no statistically significant difference between the two groups in the rate of successful maturation (Pooled risk ratio = 0.95 [0.82, 1.11], P = 0.53). Similarly, the incidence of postoperative haematoma (Pooled risk ratio = 0.73 [0.34, 1.58], P = 0.43), wound infection (Pooled risk ratio = 0.77 [0.35, 1.68], P = 0.51) and steal syndrome (Pooled risk ratio = 0.65 [0.27, 1.53], P = 0.32) were statistically comparable.

**Conclusion:**

Although more studies seem to favour the two-stage BVT approach, evidence in the literature is not sufficient to draw a final conclusion as the difference between the one-stage and the two-stage approaches for creation of a BB-AVF is not statistically significant in terms of the overall maturation rate and postoperative complications. Patency rates (primary, assisted primary and secondary) were comparable in the majority of studies. Large randomised properly conducted trials with superior methodology and adequate sub-group analysis are needed before making a final recommendation.

## Introduction

The superiority of haemodialysis (HD) access created by means of an Arteriovenous Fistula (AVF) in patients with end stage renal disease (ESRD) has been shown before. Stenosis and thrombosis is less likely to occur in a well-functioning mature AVF when compared to arteriovenous grafts (AVG) and central venous catheters (CVC), resulting in prolonged patency rates for AVFs as has been described previously [1]. Also, AVFs carry a lower risk for infection [2,3]. However, around 20%- 50% of all fistulas fail to mature into a useful HD access [4–7].

The preferred location for placing an AVF for the first time is distally at the radius, thus making it possible to place a second fistula proximally if the first one failed to mature. The order of preference for creating an AVF [8–10]:

1. Distal Radio-Cephalic
2. Proximal Radio-Cephalic
3. Brachio-Cephalic
4. Brachio-Basilic (transposed Basilic vein)

This order is in agreement with the National Kidney Foundation Kidney Disease Outcomes Quality Initiative (NKF KDOQI) guidelines [11]. However, fistulas created distally at the wrist are less likely to mature compared to proximal AVF, at the same time proximal AVF require less intervention and are likely to last longer [12]. The decision of where to create the AVF can be helped by preoperative vascular mapping using ultrasound imaging which is expected to improve chances of creating an AVF that will likely mature into a useful dialysis access [13,14]. Placement of a primary forearm fistula is feasible in 40% to 50%, with an upper arm fistula possible in an additional 25% to 35% of patients [15]. An AVF prevalence of ≥ 65% has been recommended in the KDOQI guidelines for patients undergoing HD [11], this prevalence is currently higher in Europe (67%- 91%) compared to the US (24%- 47%) [15–18]; however, the prevalence of AVFs in the US varies significantly among different dialysis units [15,19].

Dagher was the first to describe the use of basilic vein to create an AVF in the upper arm between the end of basilic vein and the side of the brachial artery to act as access for long term haemodialysis [20]. Since then, the procedure has seen several changes and modifications. Superficialisation of a brachiobasilic fistula to make it more susceptive to cannulation can be achieved either by an elevation technique without mobilisation to bring the vein superficial to the surgically reconstructed deep fascia and subcutaneous tissue in the anatomic location of the basilic vein [21], or by a transposition technique by mobilising the entire length of the basilic vein to position the vein anterolaterally through a subcutaneous flap [22].

Some of the debate surrounding brachiobasilic arteriovenous fistulas (BB-AVF) has been focused on the decision to choose between one-stage vs the two-stage techniques. The one-stage procedure aims to create a fistula between the basilic vein and the brachial artery in the upper arm in one procedure. This would require a long incision to gain access and mobilise the basilic vein making sure the anastomosis is not placed under tension and no obvious stenosis is present proximally. The main advantage of this technique is the shorter waiting time required to cannulate the fistula. Also the one-stage will prevent the patient from having to undergo another procedure and is more cost effective as hospital resources will be used only once. One of the main disadvantages of this technique is the long incision which will require a longer time to heal and also carries a higher risk for wound-related complications. Also the procedure takes longer and is more demanding [23–25]. Moreover, in a study by Anaya-Ayala et al assessing the anatomy of basilic vein found that only 66% of patients are expected to have a “normal” basilic vein entering one of two paired brachial veins close to the axilla, while up to 34% will have an “abnormal” variant that would negatively influence the newly created fistula maturation [26].

The two-stage procedure allows the basilic vein to become arterialised and as such, more resistant to torque and will become easier to mobilise in the second procedure as it gets transformed into a bigger and stronger structure. The hope is that operative difficulty and complications would be reduced with improved patency rates [27].

This review was designed to systematically assess the difference between both procedures in terms of access maturation and survival, as well as complications and interventions required to maintain patency for haemodialysis.

## Methods

This systematic review and meta-analysis were conducted according to the Preferred Reporting Items for Systematic Review and Meta-Analysis (PRISMA) guidelines [28]. No published protocol exists for this review.

### Eligibility criteria

We searched for randomised controlled trials (RCTs) and observational studies that compared the one-stage technique with the two-stage technique for creating a brachiobasilic arteriovenous fistula (BBAVF) for haemodialysis access. Case series and review articles were excluded from this review.

### Search strategy

**Fig 1:**
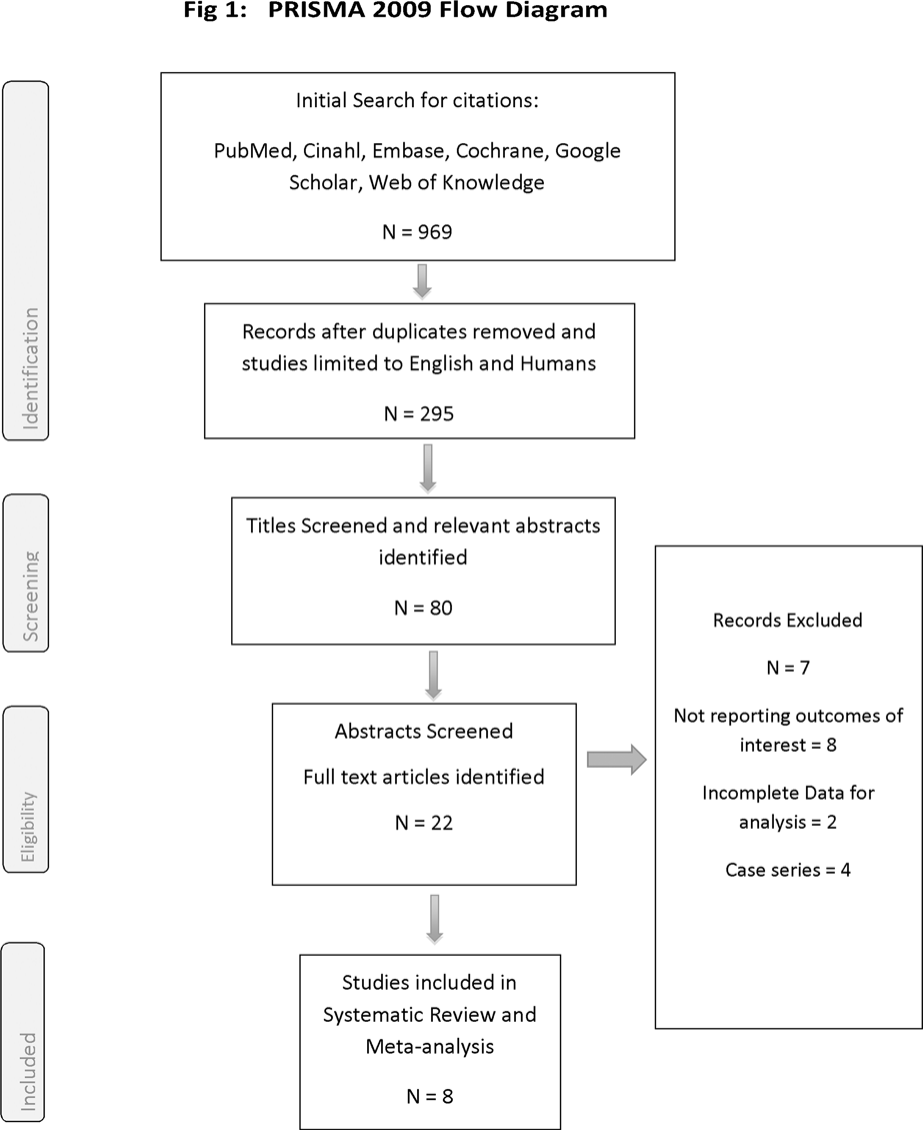
Prisma Flow Diagram. Eligibility for inclusion was determined by two researchers separately (KB, DH) by going through the abstracts of the relevant citations. Differences were settled by examining the full article by both authors, and then any remaining uncertainties regarding eligibility of studies were settled following a discussion with a third author (SRW).

A search of the literature for relevant studies was conducted in August 2014 using the following terms: ([“Basilic Vein” OR “Basilic”] AND [“Fistula” OR “Arteriovenous” OR “Access”] AND “dialysis”). We searched the online databases of: Medline, CINAHL, EMBASE, the Cochrane library and Google Scholar. We did not restrict our search by publication date or status, however, we only included studies published in English language and those conducted on humans. We also searched the bibliographies of included trials for additional studies. A summary of the study selection process can be found in the PRISMA flow diagram below [Fig. 1]. Studies were not restricted based on the duration of follow-up.

The main outcome measures for this review were successful maturation and development of postoperative complications, namely wound haematoma, wound infection and steal syndrome. Secondary outcomes were primary and secondary patency rates. Definitions for “maturation”, “primary patency” and “secondary patency” were those specified in individual studies.

### Data collection

KB and DH independently extracted the data from included studies on a Microsoft Excel spreadsheet. Any differences in recording the outcomes of interest were discussed between two authors (KB, DH), and if remained unsettled, a third author was consulted to resolve the issue (SRW). The following characteristics regarding participants were recorded: age, sex, presence of co-morbidities, primary patency rate, secondary patency rate, maturation rate and postoperative complications (Haematoma, wound infection and steal syndrome). Usability of fistula for Haemodialysis, time to first use for HD and interventions needed to maintain patency were recorded when possible. We also extracted data on compliance with the Society for Vascular Surgery (SVS) recommended standards for reports dealing with arteriovenous haemodialysis accesses [29]. To this end, we assessed whether studies provided SVS standard-based grading of factors that affect outcomes and whether studies provided SVS standard-based grading of severity of arteriovenous access complications. The studies’ inclusion and exclusion criteria were also recorded [Table 1].

**Table 1:** Characteristics of individual studies.

Study	Date published	Key aspects of design	Inclusion	Exclusion	Nature of the one stage procedure	Nature of the two stage procedure	Outcomes assessed	Main findings	Number 1 stage	Characteristics 1 stage	Number 2 stage	Characteristics 2 stage
**Vrakas** [22]	2013	Retrospective cohort study at King's College Hospital London. Mean follow up of BBAVF patients was 559 days (SD33). Median interval between first and second operations in the two stage group was 90 days. Allocation to groups was based upon preferences of the two surgeons. Patients with small veins mostly would have had 2 stage procedure.	Consecutive patients who underwent BBAVF between January 1st 2009 and December 31st 2011.	None specified	Basilic vein dissected and mobilised with preservation of the medial cutaneous nerve of the forearm. End to side arteriovenous anastomosis in the antecubital fossa.	First, BB-AVF created at the cubital fossa, then 4–6 weeks later a second procedure carried (following US assessment to determine if a second stage is necessary) for mobilisation and superficialisation of the fistula	Primary, primary assisted and secondary functional patency rates. Complications such as thrombosis, haematoma, steal syndrome, infection, venous hypertension, stenosis, mortality.	Two stage procedure patients had better functional primary, primary assisted and secondary patency rates at 1 and 2 years. Complication rates were similar.	Data were provided using number of BBAVFs as the denominator rather than the number of patients. 65 one stage procedures were performed. Number of patients was unclear.	Mean age was 58 years (SD15). 32/65 were female. 25/65 had DM. 53/65 had hypertension. Mean BMI was 29 (SD6). 29/65 were black. Mean vein size was 4.0mm (1.1SD). The only significant difference was in vein size (p = 0.041). Factors that affect outcome were not described in accordance with the SVS guidelines.	84 two stage procedures were performed. Number of patients was unclear.	Mean age was 58 years (SD15). 44/84 were female. 33/84 had DM. 67/84 had hypertension. Mean BMI was 27 (SD7). 39/84 were black. Mean vein size was 4.0mm (1.1SD). The only significant difference was in vein size (p = 0.041). Factors that affect outcome were not described in accordance with the SVS guidelines.
**Ozcan** [30]	2013	Retrospective cohort study at the authors’ institutions. Allocation to groups was based upon surgeon preference and often patients with basilic vein diameter <3mm had the 2 stage procedure. The second stage of the two stage procedure took place at 30 days. Mean follow up was for 36 months.	Patients who underwent BVT in the authors' institution(s) between January 2007 and January 2012.	None specified	Basilic vein dissected and mobilised with preservation of the medial cutaneous nerve of the forearm. End to side arteriovenous anastomosis in the antecubital fossa. HD was allowed after one month.	First, BB-AVF created at the cubital fossa, then 4 weeks later a second procedure carried for mobilisation and superficialisation of the fistula	Primary and secondary patency rates, postoperative complications such as thrombosis, haemorrhage, haematoma, infection, venous aneurysm development, mortality. Rate of fistula maturation and time to fistula maturation. Auxiliary interventions for patency.	Two stage procedure patients had a higher rate of maturation but 1 stage BVTs matured faster. Thrombosis, bleeding, haematoma incidence were lower in the two stage group. The two stage group required fewer intervention for patency within the first 10 days but after that there was no difference. Primary and secondary patency rates were better in the two stage group but no statistical analysis was performed for this outcome.	Data were provided using number of BVTs as the denominator rather than the number of patients. 47 one stage procedures on 47 patients were included and Total number of patients was 96 therefore some patients were included twice.	Mean age was 43.1 years (SD16) for men and 42.5 years (SD13) for females. 28/47 were male. Mean duration of ESKD was 63.1 months (SD17) for men and 64.5 (SD18) for women. 15/47 had hypertension. 9/47 had DM. 4/47 had heart disease. 2/47 had PVD. 9/47 were smokers. Mean basilic vein diameter was 3.46mm (SD0.2). The only significant difference between groups was in vein size (p<0.001). Factors that affect outcome were not described in accordance with the SVS guidelines.	59 two stage procedures on 59 patients were included.	Mean age was 44.9 years (SD14) for men and 44.1 (SD13) for females. 36/59 were male. Mean duration of ESKD was 61.7motnhs (SD20) for men and 63.3 (SD21) for women. 14/59 had hypertension. 11/59 had DM. 3/59 had heart disease. 3/59 had PVD. 11/59 were smokers. Mean basilic vein diameter was 2.79mm (SD0.1). The only significant difference between groups was in vein size (p<0.001). Factors that affect outcome were not described in accordance with the SVS guidelines.
**Kakkos** [31]	2010	Retrospective cohort study at Henry Ford Hospital Detroit USA on 173 consecutive patients who were scheduled for BVT. Allocation to groups was based on surgeon's preference. The length of follow up was not described explicitly although the report suggests that follow ended when fistulas were used in dialysis.	Patients who underwent BVT at the authors' institution during a 5 year period between xx and xx.	None specified	Basilic vein dissected and mobilised with preservation of the medial cutaneous nerve of the forearm. Arteriovenous anastomosis in the antecubital fossa via the brachial or proximal radial or ulnar artery. HD was allowed only after least 6 weeks.	First, BB-AVF created at the cubital fossa, then 4–6 weeks later a second procedure carried for mobilisation and superficialisation of the fistula	Maturation rates and complications such as haematomas, dehiscence, infection, steal syndrome, venous hypertension. 30 day mortality.	One stage procedures had significantly higher complication rates. Haematomas and venous hypertension occurred significantly more often in one stage procedures. Maturation rates were similar although time to first use was longer in the two stage group.	Data were provided using number of BVTs as the denominator rather than the number of patients. 76 one stage procedures were performed. One patient in the study had two distinct BVT procedures and was thus included twice but it was not clear which procedures this patient underwent.	Mean age was 59 years (SD15). 46/76 were male. 61/76 were black. 45/76 had DM. 51/76 had previous dialysis access. 6/76 were pre-haemodialysis patients. 16/76 had general anaesthesia and 60/76 were performed under local anaesthesia. The only significant baseline differences were that more patients in one stage group had a history of previous access and they also were more likely to have general anaesthesia. Notably there were no data on baseline vein diameters. Factors that affect outcome were not described in accordance with the SVS guidelines.	98 patients underwent two stage procedures.98 had the first stage and 72 subsequently underwent the second stage. One patient in the study had two distinct BVT procedures and was thus included twice but it was not clear which procedures this patient underwent.	Mean age was 62 years (SD16). 41/98 were male. 73/98 were black. 57/98 had DM. 30/98 had previous dialysis access. 14/98 were pre-haemodialysis patients. 4/98 had general anaesthesia and 94/98 were performed under local anaesthesia. Notably there were no data on baseline vein diameters. Factors that affect outcome were not described in accordance with the SVS guidelines.
**El Mallah** [32]	1998	Prospective randomised controlled trial at El Menoufia University Hospital Egypt. Allocation to groups was performed randomly and groups were matched for age and gender. No details on the randomisation process were provided. Follow up was for 6–24 months.	It involved 40 patients who were admitted for secondary vascular access procedures between June 1993 and December 1995.	None specified	BB-AVFs were made using the traditional one stage technique.	First, BB-AVF created at the cubital fossa by anastomosing a mobilised segment of basilica vein to the brachial artery. Then 2–4 weeks later a second procedure carried for mobilisation and superficialisation of the fistula.	Patency at 4 weeks and patency at end of follow up period. Aneurysm formation and infection.	Early patency was achieved in 12/20 in the one stage group versus 18/20 in the two stage group. Patency at end of follow up was 10/20 versus 16/20. The authors did not use an intention to treat analysis. When an intention to treat analysis was used, the difference was not significant. There was no significant difference in in infection or aneurysm rates.	20 patients who underwent 20 one stage procedures were included.	Mean age was 32.5 years (SD5.8). 12/20 were male. Mean period of follow up was 16 months (SD3.5). Factors that affect outcome were not described in accordance with the SVS guidelines.	20 patients who underwent 20 two stage procedures were included. One fistula occluded in the interval between stages and thus was excluded.	Mean age was 35.8 years (SD7.3). 11/20 were male. Mean period of follow up was 14.8 (SD5). Factors that affect outcome were not described in accordance with the SVS guidelines.
**Syed** [33]	2012	Retrospective cohort study on 106 patients who underwent BVT at the Methodist DeBakey Heart & Vascular Centre in Texas. Choice of one stage BVT or two stage BVT was based upon surgeon preference. Follow up was for 3 years.	It involved 106 patients who underwent BVT between June 2006 and June 2010. It is unclear whether the cases were consecutive. Data came from a computerised database.	None specified	Brachial artery tobrachial vein anastomosis along with the superficialtransposition, all in the same procedure	The anastomosis was createdin the first stage and, subsequently, the vein wastransposed in the second stage	Primary, primary assisted and secondary patency up to three years, reinterventions, mortality, major complications, fistula maturation and complications such as infections, steal syndrome,	Primary patency and assisted primary patency rates were better in the one stage group. Other outcomes were not significantly different.	29 patients underwent one stage BVT	Mean age was 54 years (SD21). 14/29 were male. 16/29 had current catheter usage at the time of the surgery. 16/29 had prior ipsilateral access. Average BMI was 28.1, 16/29 had DM, 28/29 had hypertension, 5/29 had coronary artery disease, 2/29 had congestive heart failure. 13/29 had GA and the others had regional arm block. The only significant differences in baseline characteristics between groups was in regards to history of catheter use and prior ipsilateral access procedure. Factors that affect outcome were not described in accordance with the SVS guidelines.	77 patients underwent the two stage procedure.	Mean age was 54 years (SD14. 29/77 were male. 67/77 had current catheter usage at the time of surgery. 16/77 had prior ipsilateral access. 39/77 had prior failure of an arteriovenous fistula. Average BMI was 28.1, 42/77 had DM, 71/77 had hypertension, 21/77 had coronary artery disease, 7/77 had congestive heart failure. 27/77 had GA and others had regional arm block. Factors that affect outcome were not described in accordance with the SVS guidelines.
**Agarwal** [34]	2014	Retrospective cohort study involving 144 consecutive patients who underwent BVT at a US hospital. Patients with basilic vein diameter of <4mm were chosen for the two stage procedure. Mean follow up duration was unclear. Some patients were followed for greater than 4 years.	It involved consecutive patients who underwent BVT creation between January 2005 and December 2009 and who received all access-related care (surgical and radiological) up to a 4 year follow up point in December 2013.	Patients were excluded if interventions or follow up had taken place at an outside institution.	Not specified	Not specified	Maturation rates, mean time to initiation of fistula use, intensity of percutaneous interventions per patient year on dialysis, primary patency, primary assisted patency and secondary patency annually.	Modest reduction in primary and secondary patency rates in the two stage group compared to the one stage group	61 patients underwent 61 one stage BVTs	Mean age was 59.1 years. No other were provided on baseline characteristics. Factors that affect outcome were not described in accordance with the SVS guidelines.	83 patients underwent 83 two stage BVTs.	Mean age was 61.5 years. No other data were provided on baseline characteristics. Factors that affect outcome were not described in accordance with the SVS guidelines.
**Hossny** [21]	2003	Cohort study involving 70 brachiobasilic fistulas in 70 patients at Menofia University Egypt. It is unclear whether it was prospective or retrospective although it seems to be prospective. The study compared basilic vein transposition versus a one stage elevation procedure versus a two stage elevation procedure. It is unclear on what grounds patients were selected for different procedures. Mean follow up time was 25.8 months.	It involved 70 brachiobasilic fistulas that were performed in 70 patients over an unspecified 2 year period at the author's institution.	None specified	30 fistulas created using a traditional one stage BVT to create a BB-AVF20 fistulas created in a one stage elevation technique, the basilic vein was brought superficial to the deep fascia and subcutaneous tissue rather than through a subcutaneous tunnel	20 fistulas created in a two stage elevation technique, the basilic vein was brought superficial to the deep fascia and subcutaneous tissue rather than through a subcutaneous tunnel	Ability to access fistula for dialysis, cumulative secondary patency, complications (oedema, haematoma, thrombosis, venous hypertension, lymph leakage), perioperative mortality,	The one stage BVT had a lower complication rate and was favoured by the dialysis staff compared to basilic vein superficialisation techniques	20 patients underwent 20 one stage BVTs, while 20 patients underwent one stage basilic vein elevation procedure	For the one stage BVT: Mean Age = 45.7 (16–98), 12/40 were created in male patients, 17/20 had diabetes and 10/20 had hypertensionFor the one stage basilic vein elevation: Mean age = 49.3 (26–71), 12 had diabetes and 7 had hypertension. Factors that affect outcome were not described in accordance with the SVS guidelines.	20 patients underwent two stage basilic vein procedure	Mean Age = 54 (32–71), 8 were created in male patients. Diabetics = 4/20 and 5/20 had Hypertension. Factors that affect outcome were not described in accordance with the SVS guidelines.
**Effat** [35]	2013	Cohort study involving 104 patients who underwent 106 Brachiobasilic fistulas at Zagazig University Hospital from October 2010 to December 2011. It is unclear whether it was prospective or retrospective. Comparison between one stage BVT, Two stage BVT, two stage superficialisation. Allocation to groups was based upon surgeons’ or patients' preferences. The period of follow up was not specified.	Scheduled for brachiobasilic fistula with a basilic vein >2.5mm diameter and a brachial artery >3mm.	Patients were excluded if vein diameter <2.5mm, failure of BBAVF to mature in staged groups, steel or massive venous hypertension after creation of the brachiobasilic shunt and failed to be corrected, patients who refused the second stage or who were lost to follow up between stages.	All fistulas created using a traditional one stage BVT to create a BB-AVF	38 fistulas were created using a two stage BVT technique, stage one involved forming a BB-AVF, the second stage involved mobilisation and superficialisation of the fistula. In 40 fistulas, they carried a two stage superficialisation procedures without transposing the basilic vein	functional patency (ability to access the fistula for haemodialysis), mean time to use the fistula, complications such as haematomas requiring exploration, wound dehiscence or infection, thrombosis, steal syndrome, venous hypertension requiring intervention, failure to mature.	Lower patency rates for the one stage technique and increased chance of developing postoperative complications compared to the two stage technique	28 one stage BVTs performed. Number of patients unclear.	Mean age = 43.6 ± 11.9, 13/28 were male, 13/28 had diabetes and 16/28 had hypertension. Factors that affect outcome were not described in accordance with the SVS guidelines.	38 two stage BVTs and 40 two stage superficialization. Number of patients unclear	For the two stage BVT: Mean age = 48.4 ± 10.2, 20/38 were created in male patients. 19/38 had diabetes and 22/38 had hypertensionFor the two stage elevation: Mean age 47.5 ± 8.4, 16/40 were created in male patients, 23/40 had diabetes and 26/40 had hypertension. Factors that affect outcome were not described in accordance with the SVS guidelines.

### Quality assessment for risk of bias

The Downs and Black Tool was used for quality assessment [36]. This tool consists of a total of 27 questions assessing the quality of reporting, external validity and internal validity generating scores between 0 to 32 which includes a score of 0–5 for sample size justification, however, this has been modified by awarding one point for studies that reported on sample size calculations, and zero for those that did not report a methods of sample size calculation. Hence, the modified score ranged from 0 to 27, with higher scores reflecting higher quality. Details of the quality assessment can be found in a separate supplemental table [S1 Table].

### Data analysis

Statistical analyses were performed using Review Manager version 5.3 [37]. We used the random effects model of DerSimonian and Laird [38] to calculate pooled risk ratios for categorical outcomes measures. The Cochran’s Q test was used to determine statistical heterogeneity among studies. 95% confidence interval and P-values < 5% were used to determine statistical significance. We compared between the fixed and random effects modelling to produce a sensitivity analysis aimed at detection of the influence of publication bias of small-study effects[39]. Regarding the meta-analysis we additionally, performed a sensitivity analysis limited to published articles only.

## Results

### Study selection

The results of the study selection process are summarised in the PRISMA flow diagram [Fig. 1]. We started with a total of 969 citations. Following the removal of duplicates and limiting the search criteria to studies conducted on humans and in English language, we were left with 295 citations. We then screened the titles of those papers, and found 80 potentially relevant citations. The abstracts of those titles were examined for relevant outcomes, and 22 papers were evaluated for eligibility criteria, of those 8 citations met our criteria and were included in the systematic review [21,22,30–35]. Of those 8 studies, 1 was a randomised controlled studies (RCT). Five were retrospective cohort studies [22,30,31,33,34] and 2 studies were cohort studies but it was unclear whether they were retrospective or prospective [21,35]. This last citation was a conference presentation which we included in the review, however we also ran a group of sensitivity tests excluding the data from this citation and including data extracted from published papers only [35].

Six of the included studies compared outcomes between 1-stage versus 2-stage BB-AVF formation techniques, while Hossny et al compared 3 different groups, first group of patients had traditional 1-stage basilic vein transposition (BVT), while the second group had 1-stage basilic vein elevation, and the third group underwent a 2-stage BB-AVF. For the sake of this meta-analysis, we pooled the first 2 groups from this particular study together [21]. Similarly, Effat had 3 groups of patients in his conference paper, the first group had standard 1-stage BVT, whereas the second group had a 2-stage BVT and the last group consisted of patients who had 2-stage superficialization of the basilic vein to create BB-AVF. We pooled the data from the 1-stage procedures in this last study together in the meta-analysis [35].

### Participants

The studies included a total of 849 patients who had 859 fistulas, of those 366 fistulas were formed using a 1-stage technique, while the remaining 493 fistulas were created in a 2-stage technique. Overall, 432 were male patients versus 417 female patients. Kakkos et al [31] did not specify the male to female ratio in the 72 patients who underwent a 2-stage procedure in their study, however, in the remaining studies, 181 men had 1-stage fistula procedure compared to 164 in the 2-stage group. Similarly, 226 in the 1-stage group were female patients compared to 150 in the 2-stage group. Of the 6 [21,22,30,31,33,35] studies that reported past history of diabetes, 143/295 patients were in the 1-stage group while 202/390 were in the 2-stage group. History of hypertension was reported in 5 studies [21,22,30,33,35], with 123/219 patients in the 1-stage group and 202/390 in the 2-stage group having the diagnosis. All studies reported on findings in adult patients with end stage renal disease (ESRD), El-Mallah [32] had the youngest patients (23.5 ± 5.8 years for the 1-stage group, and 35.8 ± 7.3 years for patients in the 2-stage group), while the remaining studies included patients in their fifties and sixties [Table 1]. Inclusion and exclusion criteria of studies among other characteristics are outlined in [Table 1]. Main outcomes reported in studies are summarised in [Table 2].

**Table 2:** Main outcomes from included studies.

	1-stage procedure					2-stage procedure				
Study	Number of 1-stage fistulas	Patency	Haematoma	Wound Infection	Steal	Number of 2-stage fistulas	Patency	Haematoma	Wound infection	Steal
**Vrakas** [22]	65 one stage procedures were performed. Number of patients was unclear.	Primary functional patency at 1 and 2 years was 71% and 53%. Assisted Primary functional patency at 1 and 2 years was 77% and 57%. Secondary functional patency at 1 and 2 years was 79% and 57%. The definitions for patency outcomes were based upon the SVS guidelines.	3 / 65No SVS grading was provided	3 / 65	2 / 65No SVS grading was provided	84 two stage procedures were performed. Number of patients was unclear.	Primary functional patency at 1 and 2 years 87% and 75%. Assisted Primary functional patency at 1 and 2 years was 95% and 77%. Secondary functional patency at 1 and 2 years was 95% and 77%	3 / 84No SVS grading was provided.	2 / 84	3 / 84
**Ozcan** [30]	47 one stage procedures on 47 patients were included and Total number of patients was 96 therefore some patients were included twice.	Primary patency at 1, 2 and 3 years was 33/47 (70%), 30/47 (64%), and 27/47 (54%) Secondary patency at 1, 2 and 3 years was 36/47 (76%), 43/47 (72%), and 31/47 (66%). The definitions for patency outcomes were unclear.	8 / 47No SVS grading was provided	6 / 47No SVS grading was provided	4 / 47No SVS grading was provided but all required surgical management	59 two stage procedures on 59 patients were included.	Primary patency at 1, 2 and 3 years was 41/59 (84%), 36/59 (73%), and 34/59 (69%). Secondary patency at 1, 2 and 3 years was 44/59 (90%), 40/59 (82%), and 38/59 (77%)	3 / 59No SVS grading was provided	5 / 59No SVS grading was provided	3 / 59No SVS grading was provided but all require surgical treatment
**Kakkos** [31]	76 one stage procedures were performed. One patient in the study had two distinct BVT procedures and was thus included twice but it was not clear which procedures this patient underwent.	Not reported	10 / 76No SVS grading was provided. Across the whole study, most were grade 1 or 2 and 3 were grade 3	5 / 76No SVS grading was provided	3 / 76No SVS grading was provided but all were managed conservatively	98 patients underwent two stage procedures.98 had the first stage and 72 subsequently underwent the second stage. One patient in the study had two distinct BVT procedures and was thus included twice but it was not clear which procedures this patient underwent.	Not reported	3 / 72No SVS grading was provided. Across the whole study, most were grade 1 or 2 and 3 were grade 3	0 / 72No SVS grading was provided	2 / 72No SVS grading was provided but all were managed conservatively
**El Mallah** [32]	20 patients who underwent 20 one stage procedures were included.	Early patency (4 weeks) = 12/20 (60%), Overall patency (at the end of follow-up) = 10/20 (50%), The definitions for patency outcomes were unclear.	-	3 / 20No SVS grading was provided but they were described as mild infections	0 / 20	20 patients who underwent 20 two stage procedures were included. One fistula occluded in the interval between stages and thus was excluded.	Early patency (4 weeks) 2-stage = 18/20 (90%). Overall patency at the end of the study = 16/20 (80%)	-	1 / 20No SVS grading was provided but they were described as mild infections	0 / 20
**Syed** [33]	29 patients underwent 20 one stage BVT	Primary patency at 1, 2 and 3 years was 82%, 81%, and 51%. Assisted primary patency at 1, 2 and 3 years was 91%, 77%, and 48%. Secondary patency at 1, 2 and 3 years was 91%, 80%, and 58%. The definitions are similar to those in the SVS guidelines.	2 / 29No SVS grading was provided.	0 / 29	0 / 29	77 patients underwent the two stage procedure.	Primary patency at 1, 2 and 3 years was 67%, 27%, and 18%. Assisted primary patency at 1, 2 and 3 years was 77%, 41%, and 24%. Secondary patency at 1, 2 and 3 years was 81%, 61%, and 45%	6 / 77No SVS grading was provided.	3 / 77No SVS grading was provided.	3 / 77No SVS grading was provided.
**Agarwal** [34]	61 patients underwent 61 one stage BVTs	Primary unassisted patency at 1 and 2 years was 26% and 7%. Primary assisted patency at 1, 2, 3 and 4 years was 67%, 38%, 21% and 8%. Secondary patency at 1, 2, 3 and 4 years was 86%, 75%, 69% and 57%. The definitions are similar to those in the SVS guidelines.	-	-	-	83 patients underwent 83 two stage BVTs.	Primary unassisted patency at 1 and 2 years was 13% and 0%. Primary assisted patency at 1, 2, 3 and 4 years was 66%, 39%, 7% and 0%. Secondary patency at 1, 2, 3 and 4 years was 76%, 71%, 49% and 25%	-	-	-
**Hossny** [21]	20 patients underwent 20 one stage BVTs, while 20 patients underwent one stage basilic vein elevation procedure	The study reported 87% cumulative secondary patency rate at 1 year across all groups, with 86.7% for the BVT group, 90% for the 1-stage elevation group and 84.2% for the 2-stage elevation group. 1 death was excluded from final analysis. Cumulative secondary patency rate at 2 years for all groups was 75%, with 82.8& for the BVT group, 70% for the 1-stage elevation group and 68.4% for the 2-stage elevation group. 2 deaths were excluded from final analysis.	6 / 50No SVS grading was provided.	-	0 / 50	20 patients underwent two stage basilic vein procedure	The study reported 87% cumulative secondary patency rate at 1 year across all groups, with 86.7% for the BVT group, 90% for the 1-stage elevation group and 84.2% for the 2-stage elevation group. 1 death was excluded from final analysis. Cumulative secondary patency rate at 2 years for all groups was 75%, with 82.8& for the BVT group, 70% for the 1-stage elevation group and 68.4% for the 2-stage elevation group. 2 deaths were excluded from final analysis.	5 / 20No SVS grading was provided.	-	0 / 20
**Effat conference** [35]	28 one stage BVTs performed. Number of patients unclear.	Not reported	2 /28No SVS grading was provided.	2 / 28No SVS grading was provided	2 / 28No SVS grading was provided	38 two stage BVTs and 40 two stage superficialization. Number of patients unclear	Not reported	7 / 78No SVS grading was provided	12 / 78No SVS grading was provided	0 / 78No SVS grading was provided

### Successful maturation rate

**Fig 2:**
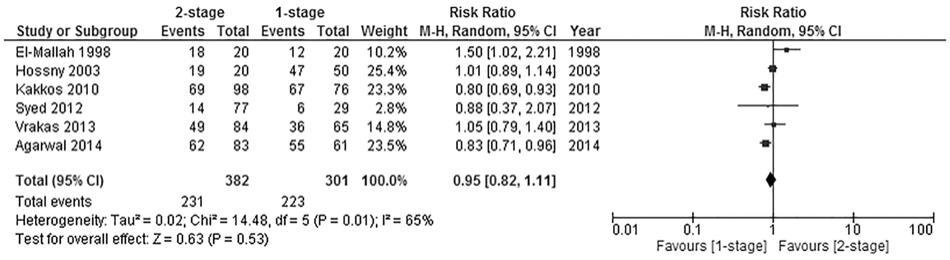
Successful maturation rate.

Successful maturation rates were reported in 6 of included studies [21,22,31–34]; the criteria used for reporting maturation are found in [Table 3]. Those studies had a combined total of 683 fistulas, 301 of those were created in the one stage group, whereas 382 were created in the two stage group. The difference between the two groups was not significant in pooled analysis (Pooled risk ratio = 0.95 [0.82, 1.11], 95% CI, P = 0.53) [Fig. 2]. Heterogeneity was detected statistically (Cochran’s Q = 14.48; degree of freedom (DF) = 5; P = 0.001; I_2_ = 65%). The significance of the results was not altered when using the fixed effects analysis model as a sensitivity test to detect publication bias (Pooled risk ratio = 0.92 [0.84, 1.01], 95% CI, P = 0.07).

**Table 3:** Maturation.

Study	One stage	Two stage	Source of data	comments
Vrakas [22]	36 / 65	49 / 84	Primary failure rates were reported. This was defined as an AVF that was never used for dialysis. Primary failure may have resulted from inadequate maturation, early thrombosis, failure of first cannulation, and other complications which made AVF unusable. Successful maturation rates were derived from these data.	The number of AVFs that required intervention to assist maturation is unclear.
Kakkos [31]	67 / 76	69 / 98	Maturation rates were reported. Maturation was based upon clinical judgement (development of basilic vein dilatation and thrill for a sufficient length).	Includes fistulas that required intervention to assist maturation for dialysis. 7 one stage fistulas required such intervention and 3 two stage fistulas required such intervention.
El-Mallah [32]	12 / 20	18 / 20	Patency at 4 weeks was reported and we used this figure to determine successful maturation. The authors did not provide a definition for patency.	The number of AVFs that required intervention to assist maturation is unclear.
Syed [33]	6 / 29	14 / 77	Maturation rates were reported. Fistula maturation was defined as dilation of the vein to allow cannulation and support dialysis at a minimum flow rate of 350ml/min for at least 3 sessions.	The number of AVFs that required intervention to assist maturation is unclear.
Agarwal [34]	55 / 61	62 / 83	Maturation rates were reported. Maturation was defined as the use of the fistula for haemodialysis for any amount of time or, if it was not used, documentation in surgical or renal records that the fistula was mature and ready for use based upon successful cannulation and/or physical examination by vascular surgery.	Includes an unspecified number of fistulae that needed percutaneous intervention to assist maturation.
Hossny [21]	47 / 50	19 / 20	Numbers of fistulas that were successfully used for dialysis at 6 weeks were reported.	No patients needed reintervention to assist achievement of successful dialysis at 6 weeks.

### Postoperative complications

#### Haematoma

**Fig 3:**
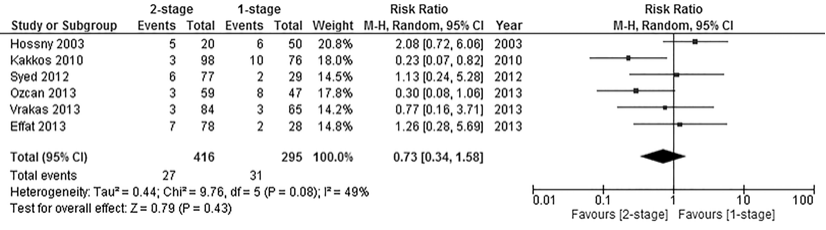
Postoperative Haematoma.

The incidence of postoperative wound haematoma was reported in 6 of the included studies [21,22,30,31,33,35] with a total of 711 fistulas, of those, 295 fistulas were created in the 1-stage group and 416 fistulas in the 2-stage group. Analysis of pooled data showed the difference was not significant (Pooled risk ratio = 0.73 [0.34, 1.58], 95% CI, P = 0.43) [Fig. 3]. Heterogeneity was not detected statistically (Cochran’s Q = 9.76; degree of freedom (DF) = 5; P = 0.08; I_2_ = 49%). The results were not changed significantly when using the fixed effects analysis model as a sensitivity test to detect publication bias (Pooled risk ratio = 0.67 [0.41, 1.11], 95% CI, P = 0.12). A sensitivity test by excluding the data from the conference paper by Effat [35] was carried out, and no significant difference was found in the incidence of postoperative haematoma between the two groups (Pooled risk ratio = 0.67 [0.27, 1.64], 95% CI, P = 0.38).

#### Wound infection

**Fig 4:**
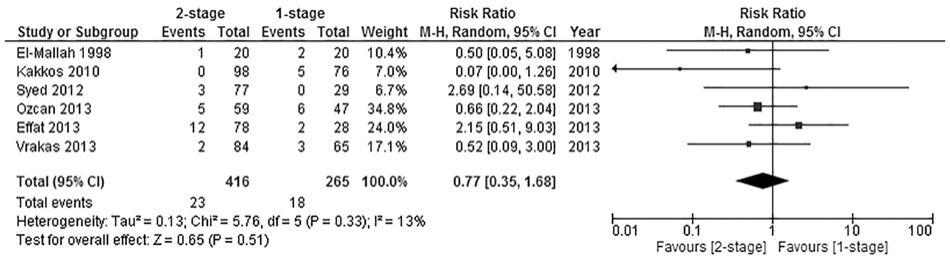
Postoperative wound infection.

Six of the included studies [30–33,35] reported on the incidence of postoperative wound infection with a total number of 681 fistulas, 265 of those belonged to the 1-stage group, while 416 consisted of 2-stage fistulas. Meta-analysis of the pooled data showed the difference between groups not to be significant (Pooled risk ratio = 0.77 [0.35, 1.68], 95% CI, P = 0.51) [Fig. 4]. There was no evidence of statistical heterogeneity (Cochran’s Q = 5.76; degree of freedom (DF) = 5; P = 0.51; I_2_ = 13%). The results were not changed significantly when using the fixed effects analysis model (Pooled risk ratio = 0.73 [0.39, 1.37], 95% CI, P = 0.32). A sensitivity test by excluding the data from the conference paper by Effat [35] was carried out, and no significant difference was found in the incidence of postoperative wound infection between the two groups (Pooled risk ratio = 0.57 [0.25, 1.27], 95% CI, P = 0.17).

#### Steal syndrome

**Fig 5:**
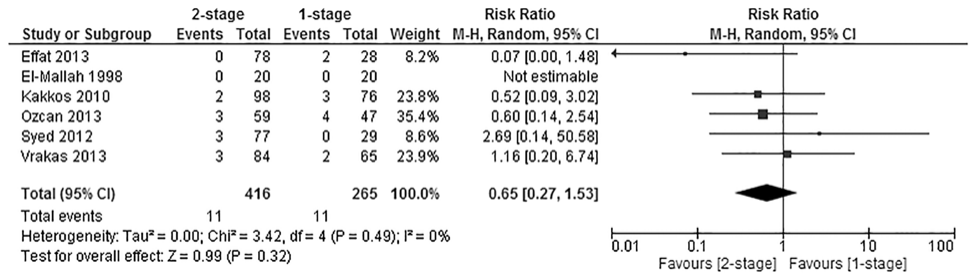
Steal syndrome.

Six of the studies [22,30–33,35] reported on the risk of developing significant postoperative ischaemia (steal syndrome). Those studies had a combined total of 681 patients, of those, 265 belonged in the 1-stage group, while 416 belonged in the 2-stage group. Analysis of pooled data showed the difference was not significant (Pooled risk ratio = 0.65 [0.27, 1.53], 95% CI, P = 0.32) [Fig. 5]. Heterogeneity was not detected statistically (Cochran’s Q = 3.42; degree of freedom (DF) = 4; P = 0.49; I_2_ = 0%). The results were not changed significantly when using the fixed effects analysis model (Pooled risk ratio = 0.64 [0.29, 1.40], 95% CI, P = 0.26). A sensitivity test by excluding the data from the conference paper by Effat [35] was carried out, and no significant difference was found in the incidence of postoperative haematoma between the two groups (Pooled risk ratio = 0.79 [0.32, 1.94], 95% CI, P = 0.60).

## Systematic Review

El-Mallah in his paper titled “Staged basilic vein transposition for dialysis angioaccess” published in 1998 [32] compared outcomes in two groups randomly allocated to receive either 1-stage BVT or 2-stage BVT. The difference in early patency rates was significant and favoured the 2-stage approach (60% of 1-stage vs 90% of 2-stage, P < 0.05), as well as overall patency rates at the end of follow-up (50% of 1-stage vs 80% of 2-stage, P < 0.05) [32]. Postoperative wound infection rate also favoured the 2-stage approach with one case compared to 3 in the 1-stage group. There was no difference in postoperative aneurysmal dilatation, and there was no significant ischaemia (steal syndrome) reported in either of the two groups [32].

Hossny looked at the different surgical techniques used in creation of a BB-AVF in 2003 [21]. He compared patency rates and dialysis related complications in 70 patients divided in 3 groups, 30 of those patients had traditional BVT whereas 20 patients had 1-stage BB-AVF with elevation and the remaining 20 patients had 2-stage fistula with elevation of the vein [21]. Cumulative secondary patency rates were comparable among the 3 groups at 1 year and 2 years; at 1 year (86.7% for the BVT group, 90% for the 1-stage elevation group and 84.2% for the last group), while at 2 years (82.8%, 70%, and 68.4%, respectively) [21]. Similarly, no significant difference was found in his study in postoperative early thrombosis across the groups. Postoperative arm oedema occurred in a total of 14 patients, all of whom had temporary subclavian access catheters sited on the same arm on which the new fistula was created. All 14 cases were managed conservatively with success. The difference in developing postoperative haematoma significantly favoured the traditional BVT approach compared to the two elevation methods with fewer haematomas reported in the first group. Interestingly, the dialysis staff were more satisfied with the 1-stage BVT technique, whereas only 53.3% reported satisfaction with the elevation technique (1-stage and 2-stage techniques) (P < .001) [21].

Kakkos et al also tried to answer the question of “What is the Optimal Technique” for performing a BVT in a retrospective study of 173 patients published in 2010 [31]. They found that the incidence of venous hypertension (17% vs 4%, P = 0.004), wound infection (13% vs 3%, P = 0.012) and all complications (43% vs 11%, P < 0.001) were significantly higher in patients who had 1-stage BVT when compared to those who had a 2-stage BVT [31]. Time to fistula use in HD was—expectedly—shorter in the 1-stage group (Median = 68 (49–103) days) compared to the 2-stage group (Median = 132 (102–166) days). This difference was significant (P < 0.001) [31]. However maturation rates were similar (85% for the 1-stage groups versus 82% for the 2-stage group, P = 0.49). Median time to use the fistulas for HD in the 2-stage group was 66 in patients who developed postoperative complications, compared to 50 days in those who did not (P = 0.019). They also found that wound infection occurred more in patients who were operated under general anaesthetic compared to those who had their procedures done under local anaesthetic (OR 38, P < 0.001). Also, venous hypertension was found to occur more frequently in patients who developed postoperative wound haematoma, but the difference was not statistically significant (18% vs 6%, P 0.12) [31]. A trend was noted towards steal being more common in patients with previous vascular access than in those who did not have such access (4.9% vs 1.1%, P = 0.19) [31].

Syed et al carried out a similar study comparing the outcomes of 1-stage (29 patients) and 2-stage (77 patients) BVT and published their findings in 2012 [33]. 79% of patients in the 1-stage group had a history of a previously failed access for HD compared to 51% of the 2-stage group. They found that the rate of primary failure was comparable between both groups (21% vs 18%) [33]. In their study, patients who had 1-stage BVT had better patency rates when compared to those who had 2-stage procedures at 1 year (82% vs 67%), 2 years (81% vs 27%) and 3 years (51% vs 18%) respectively. The same finding was reported for secondary patency (91%, 80% and 58% for 1-stage BVT compared to 81%, 61% and 45% for the 2-stage group at 1, 2 and 3 years respectively) [33]. Reintervention rate in this study was 62% for the 1-stage vs 66% for the 2-stage group. It is worth noting that 87% of the patients in the 2-stage group were using catheters for dialysis, whereas 55% of the 1-stage group were dialysing through a catheter at the time of access formation [33].

Ozcan et al published a paper in 2013 with preliminary results from their study comparing 1-stage and 2-stage BVT to create AVF access in HD patients [30]. They retrospectively divided their patients to those with a basilic vein > 3 mm and who had a 1-stage BVT procedure, and those with a basilic vein < 3 mm who had a 2-stage procedure. Although the diameter of the basilic vein was statistically higher in the first group (3.46 ± 0.2 mm) compared to the second group (2.79 ± 0.1 mm) (P < 0.005), the rate of fistula maturation was significantly lower in the first group (66% vs 77%, P < 0.005) [30]. Also, postoperative complications were significantly higher among the first group of patients who had 1-stage BVT. Thrombosis occurred in 34% compared to 23% of patients who had a 2-stage procedure, haemorrhage in (36% vs 14%) and haematoma in (17% vs 6%) respectively. Time required for the fistula to mature was significantly shorter in the first group (Mean 41 ± 14 days) compared to the second group (Mean 64 ± 28 days) (P < 0.05) [30]. Early interventions (≤ 10 days) for fistula thrombosis occurred more frequently in the first group (21% vs 12%, P < 0.05), although there was no significant difference in terms of late interventions (≥ 10 days) required to deal with access thrombosis (20% in the first group vs 22% in the second) [30]. Also they reported superior primary patency rates at 6, 12, 18, 24, 30 and 36 months for those who had 2-stage BVT fistulas compared to the first group of patients (1-stage 83%, 70%, 68%64%, 60% and 57% versus 88%, 84%, 80, 73%, 71% and 69% for the 2-stage respectively). Similarly, the 1-stage had lower secondary patency rates at 6, 12, 18, 24, 30 and 36 months when compared to the 2-stage group (85%, 76%, 74%, 72%, 70% and 66% versus 94%, 90%, 84%, 82%, 80% and 77% respectively) [30].

Similarly, Vrakas et al evaluated the difference in outcomes between 1-stage (65 fistulas) and 2-stage (84 fistulas) BB-AVFs performed in 141 patients [22]. They performed ultrasound scans 4–6 weeks after the first stage procedure to determine if a second stage was required. Patients who had their fistulas created in a 1-stage approach had a bigger preoperative basilic vein diameter (4.0 ± 1.1 mm vs 3.6 ± 1.3 mm, P = 0.041) [22]. There was no difference in primary failure between the groups (45% vs 42%, P = 0.718), however the 1-stage BB-AVF had significantly lower primary (71% vs 87%; P = 0.034), assisted primary (77% vs 95%; P = 0.017), and secondary (79% vs 95%; P = 0.026) functional patency rates compared to the 2-stage BB-AVF [22]. Multivariate Cox regression analysis showed that the 1-stage procedure was 3.2 times more likely to fail (P = 0.028), and male gender was associated with loss of access (P = 0.054). 66% of the fistulas in the first group were used successfully for HD compared to 60% in the 2-stage group (P = 0.407), and intervention before first successful HD session was equivalent between both groups (21% vs 11%, P = 0.201) [22]. Overall, 93 (62%) fistulas were successfully used for HD (66% 1-stage vs 60% 2-stage; P = 0.407), of the remaining 56 (38%), 19 fistulas (34%) failed before needling, 2 (4%) received a renal transplant, 7 (13%) died, and 28 (50%) BBAVFs remain patent in patients awaiting to start HD [22].

Agarwal et al examined the outcomes of 1-stage vs 2-stage BVT AVF. They included patients who underwent percutaneous angioplasty (assisted maturation) in calculating the overall maturation rate which was 90% for the 1-stage group (55/61 patients) compared to 75% of the 2-stage group (62/83 patients) (P = 0.02). Subgroup analysis showed that both men (54/66 patients) and women (64/78) in this study had a maturation rate of 82% (P = 0.97)[34]. Primary unassisted patency rates were comparable between the groups (69%, 52%, 26%, and 7% for the 1-stage BVT at 3 months, 6 months, 1 and 2 years; compared to 58%, 35%, 13%, and 0% of the 2-stage group, respectively (p = 0.12) [34]. Similarly, no significant difference was found in secondary patency on an intent to-treat basis (86%, 75%, 69%, and 57% at 1, 2, 3, and 4 years for 1-stage group; compared to 76%, 71%, 49%, and 25% of the 2-stage group, respectively); (p = 0.12) [34]. The intensity of percutaneous interventions in their study was 1.84/patient-year of dialysis (PYD) for the 1-stage group versus 2.15/PYD for the 2-stage group (P = 0.57) [34]. They suggested that although the 2-stage BB-AVF technique resulted in modest reduction in maturation and patency rates, it should still be favoured to the use of synthetic grafts in patients who would not be suitable for a 1-stage BB-AVF procedure [34].

The number of AVFs that failed to progress from the first stage to the second stage in the two-staged BVT approach were unclear in four studies [22,30,34,35]. In the remaining four, El-Mallah [32] reported 1/20 patient which had an occluded shunt, while Hossny [21] also had 1/20 patient failing to progress due to spontaneous thrombosis within the first 4 weeks postoperatively. Syed [33] had 2/77 patients that never progressed to the second stage of the procedure. Kakkos [31] reported that 26/98 of his patients never had a second stage procedure (thrombosed (n = 4), failed to mature and was abandoned during the re-exploration (n = 12), patient refused the procedure (n = 3), lost to follow-up (n = 1), died (n = 2), venous hypertension (n = 2), venous monomelic neuropathy (n = 1) requiring ligation, moved out of state (n = 1)).

Number of interventions required to maintain patency or to improve the fistula maturation rates were not reported clearly in all studies. Hossny reported that in the one-stage group one patient underwent ligation and another had a surgical revision, same numbers occurred in the two-stage group. All ligations were done to treat venous hypertension, whereas all revisions were performed to improve poor flow [21]. Kakkos reported that in the two-stage group 6 patients had endovenous angioplasty interventions, 3 had surgical revisions compared to 3 patients and 1 patient in the one-stage group respectively [31]. Syed et al performed 37 angioplasty interventions, 9 surgical revisions and 5 thrombectomy procedures in their two-stage group, compared to 14, 2 and 2 patients respectively [33]. The remaining studies either did not report data related to fistula salvage procedures or it was reported in poor details making it difficult to quantify those interventions.

With the exception of the studies by El-Mallah et al [32] and the one by Vrakas et al [22] which both reported significantly superior patency rates in the two-stage groups, and the paper by Syed [33] which conversely reported a significantly better patency rates in the one-stage BVT group, the remaining studies all reported comparable patency rates [21,30,31,34,35] [Table 2]. However it is important to point out that patency rate data were reported as percentages with the lack of clearly identifiable denominators in the majority of those studies, thus making pooling those data in a meta-analysis not feasible. Also, definitions used in individual studies included in this review for patency rates (primary, assisted primary and secondary) differed significantly.

## Discussion

The number of patients with end stage renal disease (ESRD) requiring haemodialysis (HD) is steadily rising, a trend that is expected to continue [2]. A well-functioning AVF is superior to grafts and central catheters in providing access for haemodialysis efficiently and at the same time with the least rate of access related complications. This has lead vascular surgeons to resort to the basilic vein which by virtue of its anatomical position is less likely to be damaged by repeated cannulation as with the more superficial veins of the arm and forearm. However, a consensus on how to form a brachiobasilic AVF does not exist as some surgeons choose to do this in a one-stage operation, while others prefer a two-stage procedure with the first procedure usually involving making the anastomosis between the basilic vein and the brachial artery, while in the second stage the arterialised vein is mobilised and brought closer to the skin surface to facilitate cannulation for HD sessions.

This review identified eight studies [21,22,30–35], including data from a conference paper [35] in order to increase the rigour of the review. The pooled data referred to 849 patients with a total of 859 fistulas, 366 of those fistulas belonged to patients who underwent a 1-stage BB-AVF, while 493 fistulas were performed using a 2-stage technique to create the access. The data from 6 of the included studies [21,22,30,31,33,35] were used to compare the difference between the two groups in developing postoperative haematoma which was not significant (Pooled risk ratio = 0.69 [0.30, 1.56], 95% CI, P = 0.37). Excluding the data from the conference paper by Effat [35] in a sensitivity test did not alter the result (Pooled risk ratio = 0.61 [0.23, 1.60], 95% CI, P = 0.31).

Incidence of postoperative wound infection was reported in five studies [30–33,35], and the difference between the 1-stage group and the 2-stage group was not found to be significant (Pooled risk ratio = 0.82 [0.31, 2.18], 95% CI, P = 0.69). This remained unchanged when excluding the data by Effat [35] in a sensitivity test (Pooled risk ratio = 0.57 [0.21, 1.51], 95% CI, P = 0.27).

Similarly, the difference between the two groups was not found to be significant when it came to postoperative ischaemia (steal syndrome) in the 6 studies which reported this complication [22,30–33,35] (Pooled risk ratio = 0.51 [0.20, 1.30], 95% CI, P = 0.16). We performed a sensitivity test by excluding the data by Effat [35] from the pooled data, and the result was not altered (Pooled risk ratio = 0.63 [0.23, 1.69], 95% CI, P = 0.35).

Ozcan et al [30] allocated patients to groups based on vein diameter, with those with basilic vein > 3 mm receiving a 1-stage BVT, while patients with basilic vein < 3 mm received a 2-stage BVT. Even with this seemingly advantageous difference in favour of the 1-stage approach, they reported superior patency rates and maturation rates in patients who had a 2-stage procedure with primary patency at 1, 2 and 3 years for the 1-stage group of (70%), (64%), and (54%) versus (84%), (73%), and (69%) in the 2-stage group. Secondary patency rates at 1, 2 and 3 years for the 1-stage group were (76%), (72%), and (66%) versus (90%), (82%), and (77%) in the 2-stage group.

Similarly, Vrakas et al reported a smaller mean vein diameter of (3.6 ± 1.3 mm) for the 2-stage, versus (4.0 ± 1.1 mm) fistulas created in the 1-stage group, yet their results favoured the 2-stage approach with primary functional patency at 1 and 2 years for the 1-stage group of 71% and 53% versus 87% and 75% in the 2-stage group. Assisted Primary functional patency at 1 and 2 years for the 1-stage group was 77% and 57% versus 95% and 77% in the 2-stage group, while secondary functional patency at 1 and 2 years for the 1-stage group was 79% and 57% versus 95% and 77% in the 2-stage group.

Conversely, in study by Agarwal et al [34], their patients in the 1-stage group achieved better maturation rate than those who had a 2-stage BVT fistulas (90% vs 75%, P = 0.02). They did not include any analysis between the two groups based on vein diameter. Vein diameter has been shown to negatively influence maturation and patency rates in AVFs, and is one of the main predictors of those outcomes in fistulas [40,41], and indeed has been shown to be the only independent predictor of maturation in some studies[42,43]. Syed et al reported similar findings with better primary and cumulative patencies in the 1-stage group with primary patency at 1, 2 and 3 years (82% vs 67%), (81% vs 27%) and (51% vs 18%), while (secondary patency at 1, 2 and 3 years (91%, 80% and 58% for 1-stage BVT and 81%, 61% and 45% for the 2-stage group. Variations in vein diameter between the two groups were not reported in this study.

Kakkos et al [31] did not find a significant difference in maturation between the two groups, as 15% of fistulas in the 1-stage group did not mature, compared to 18% in the second group (P = 0.49). They did however find significant difference in developing postoperative haematoma (13% vs 3%, P = 0.012), venous hypertension (17% vs 4%, P = 0.004) and overall complications (43% vs 11%, P < 0.001), all in favour of the 2-stage BVT technique.

Kim et al compared the 2-stage approach to all other AVF procedures including 1-stage BVT, radiocephalic and brachiocephalic fistulas. All of the 2-stage BB-AVFs in their study successfully matured compared to a pool consisting of all different types that showed a combined maturation rate of 52% (P = 0.001). Fistula failure occurred in 7% of the 2-stage BVT compared to 59% of other fistulas (P = 0.001), and more 2-stage BVT fistulas were used successfully for HD compared to all other fistula types (87% vs 48%, P = 0.024). Also, the patency rate at 1 year was superior in the 2-stage group compared to other AVFs (91% vs 47%, P = 0.003).

One of the limitations of this review is the low number of randomised trials included—1 study was randomised—while the remaining 7 were cohort studies. Most of these studies were retrospective. Another limitation is the variation in surgical approaches, those variations include technical differences in performing the procedure, as well as differences in equipment used and expertise among participating surgeons. Those limitations can be addressed by conducting a large randomised multi-centre trial that would adhere to a rigid protocol in patients’ selection process and performing the procedures. Another limiting factor is the lack of sufficient sub-group analysis among included studies, particularly analysis taking into account factors that are known to be associated with fistula maturation such as vein diameter. Finally, we highlight that included studies were not compliant with SVS reporting recommendations regarding baseline factors that affect outcomes or severity of complications.

## Conclusion

Although more studies seem to favour the 2-stage BVT approach, evidence in the literature is not sufficient to draw a final conclusion as the difference between the 1-stage and the 2-stage approaches for creation of a BB-AVF is not statistically significant in terms of the overall maturation rate and postoperative complications. Patency rates (primary, assisted primary and secondary) were comparable in the majority of studies. Large randomised properly conducted trials with adequate sub-group analysis are needed before making a final recommendation. Future studies should aim for compliance with established reporting standards.

## Supporting Information

S1 PRISMA Checklist PRISMA 2009 Checklist. (DOCX)

S1 Table Quality assessment score of individual studies. (DOCX)
